# Characterizing the myeloid and lymphoid immune response in a porcine model of pulmonary ischemia-reperfusion injury through flow cytometry

**DOI:** 10.1371/journal.pone.0344691

**Published:** 2026-05-21

**Authors:** Allen Duong, Andrea Mariscal, Lindsay Caldarone, Chun Xu, Lei Huang, Rayoun Ramendra, Shaf Keshavjee, Mingyao Liu, Stephen Juvet, Tereza Martinu

**Affiliations:** 1 Toronto Lung Transplant Program, University Health Network, Toronto, Ontario, Canada; 2 Toronto General Hospital Research Institute, University Health Network, Toronto, Ontario, Canada; 3 Institute of Medical Science, University of Toronto, Toronto, Ontario, Canada; 4 Ajmera Transplant Centre, University Health Network, Toronto, Ontario, Canada; 5 Department of Surgery, University of Toronto, Toronto, Ontario, Canada; 6 Department of Physiology, University of Toronto, Toronto, Ontario, Canada; 7 Division of Respirology, Department of Medicine, University of Toronto, Toronto, Ontario, Canada; University of Arizona, College of Medicine-Phoenix, UNITED STATES OF AMERICA

## Abstract

Pulmonary ischemia-reperfusion injury (IRI) is a major cause of primary graft dysfunction in lung transplantation. Porcine models better simulate physiological conditions and are important for pre-clinical studies; however, comprehensive immune assessment of porcine lungs in IRI has not been performed. We aimed to evaluate immune cells and activation states in porcine IRI models and hypothesized that myeloid and lymphoid cells would infiltrate and activate following IRI. Two sets of porcine orthotopic lung transplants were performed: a 4 h reperfusion (n = 7) and a 72 h survival model (n = 6). Both were compared to a control group without lung injury (n = 6). Lung samples were processed into single cell suspensions and cryopreserved. Thawed samples were stained with anti-porcine antibodies and analyzed by flow cytometry. Absolute counts of neutrophils and CD14^+^ monocytes increased in the allograft at 4 h and remained stable over 72 h post-transplant. CD14^-^CD163^+^ monocytes and conventional dendritic cells continued to increase by 72 h post-transplant. Lymphoid cell numbers were unchanged overall, but T cells showed increased CD25 expression and a memory phenotype at 4 h. Our analysis revealed early myeloid cell infiltration post-IRI which developed into increased inflammatory and antigen-presenting cell populations by 72 h post-transplant. A transient rise in T cell activation markers was noted, consistent with rodent models. Our findings contribute to our understanding of immunological events in porcine pulmonary IRI, a model that better mimics the clinical setting. Our flow cytometry panels allow for improved immunologic analyses of porcine models in preclinical transplantation research.

## Introduction

Animal transplant models are critical for preclinical lung transplantation research. Rodents are invaluable for studying the immune mechanisms involved in acute and chronic pulmonary allograft rejection through various models [1]. The low cost of murine models, coupled with the broad availability of murine-specific reagents, genetic tools, transgenic mice, and strain combination make them attractive. However, the genetic and physiological gap between rodents and humans limit the applicability of small animal models [1,2]. A large animal model is therefore necessary for the translation of preclinical lung transplantation research into clinical practice.

Humans share more anatomical and physiological similarities with pigs than rodents [3,4]. These similarities allow for a pig lung transplant model to mimic clinical lung transplant in many respects, such as the development of primary graft dysfunction (PGD) early post-transplant [5]. PGD, the clinical manifestation of acute lung injury early after transplantation, is characterized by progressive hypoxemia and alveolar infiltrates with diffuse pulmonary edema, and contributes to lung transplant morbidity and mortality [6,7]. Clinical classification of PGD relies on radiographic findings and PaO2/FiO2 (P/F) ratio, which are technically challenging to obtain in murine models. Typically, ischemia-reperfusion injury (IRI) is a major risk-factor for PGD and causes an acute inflammatory response in which both the innate and adaptive immune systems are substantially activated [8–10]. *In vitro* studies and rodent models show involvement of alveolar macrophages (AMΦ) as initiators of IRI through toll-like receptor 4 activation [8,11–14]. Neutrophil and monocyte infiltration and activation is also well documented in lung IRI, causing tissue injury by exacerbating ischemia-induced cell damage and death [8,15–17]. The role of the adaptive immune system in IRI is less well understood, but the involvement of T helper cells and B cells through antigen-independent mechanisms has been established in mouse models [10,18–20]. Altogether, significant changes to leukocyte activation and infiltration are involved in IRI, yet most of these findings have been derived from *in vitro* studies and rodent models.

In addition to anatomical and physiological similarities, humans share more immune-related genes and immune cells functionalities with pigs compared to rodents [3,4,21–23]. Porcine neutrophils make up a similar proportion of peripheral blood cells compared to humans and can also produce defensins [24]; similar to humans, porcine macrophages respond to toll-like receptor 9 stimuli and produce interleukin (IL)-10 [24,25]. Despite these immunological similarities, there is a lack of critical immunological tools to comprehensively assess the immune responses in pigs. Therefore, to further investigate immune changes in a porcine lung transplant model and allow for further assessment of IRI-directed therapies in a pre-clinical setting, novel porcine-specific flow cytometric panels are needed. Flow cytometry is a powerful technology for cellular immunophenotyping and allows for cell isolation through fluorescence-activated cell sorting (FACS), both of which are valuable tools to assess immune responses and activity [26].

The infiltration of myeloid and lymphoid cells observed in rodent pulmonary IRI models have not been confirmed in pigs. We hypothesized that we would be able to observe similar processes in a porcine model of lung transplantation with prolonged graft storage to induce IRI. To do this, we used two porcine lung transplant models: a 4 h reperfusion and 72 h survival model which emulates the development of PGD and assessed changes in intra-graft immune cell numbers and their activation. The development of porcine immunological tools will further enhance the value of porcine lung transplant models in the development and evaluation of pre-clinical therapies in transplantation.

## Materials and methods

### Animals

Twenty-five to 30 kg male Yorkshire domestic pigs (Caughell Farms Ltd., Fingal, Canada) were used as donors and recipients. Pigs were delivered to the Toronto General Hospital Research Institute’s Animal Resources Center animal facility a week in advance to allow for acclimatization and had daily interactions with researchers. Animals received care by Animal Resources Center staff in accordance with the Canadian Council on Animal Care. The experimental protocol was approved by the animal care committee at the Toronto General Hospital Research Institute (animal utilization protocols 2446 and 5983).

### Lung transplantation procedures and models

Orthotopic left single-lung transplantation was performed as previously described [5]. Donor pigs were sedated with ketamine (20 mg/kg, intramuscularly (i.m.)), midazolam (0.3 mg/kg, i.m.) and atropine (0.04 mg/kg, intravenously (i.v.)) and anesthesia induced with inhaled isoflurane (3–5%) with 2–3 L/min oxygen via a face mask, and maintained with propofol (5–8 mg/kg/h, i.v.) and remifentanil (2–20 µg/kg/h, i.v.) infusions. Donor pigs were sacrificed via exsanguination through aortic transection while under anesthesia, and lungs harvested immediately after exsanguination. Lungs were preserved in low-potassium dextran glucose solution (Perfadex; XVIVO, Mölndal, Sweden) and stored at 4 °C for 24 h of cold ischemia.

**Fig 1:**
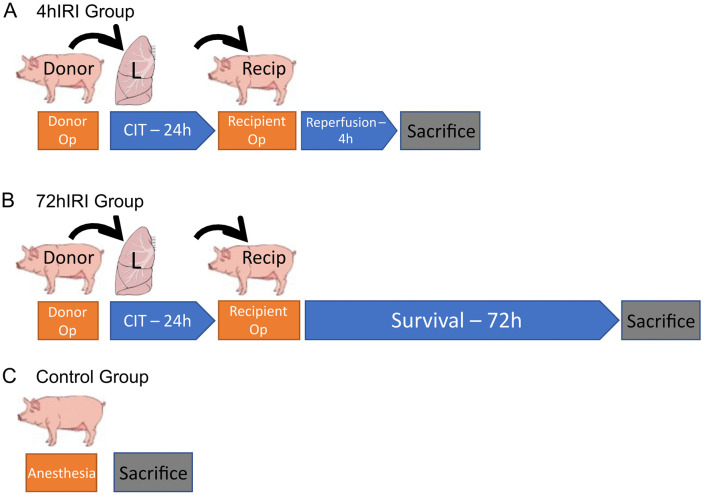
Timeline of porcine orthotopic left lung transplant models. **(A)** 4 hour reperfusion model (4hIRI). Donor operation was performed, and lungs underwent 24 hours of cold ischemic time for prolonged cold preservation. Left lung orthotopic lung transplantation was performed on recipient pig. After 4 hours of reperfusion, the pig was sacrificed. **(B)** 72 hour survival model (72hIRI). Donor operation was performed, and lungs underwent 24 hours of cold ischemic time for prolonged cold preservation. Left lung orthotopic lung transplantation was performed on recipient pig. After 72 hours post-transplant, the pig was sacrificed. **(C)** Control group (Control). Pig underwent anesthesia and was sacrificed. CIT: cold ischemic time. Op: operation. L: left. Recip: recipient.

Recipient operation was performed on two experimental groups: 4 h of reperfusion before sacrifice (4hIRI, n = 7), and 72 h of survival before sacrifice (72hIRI, n = 6) (Fig 1A-B). The 4 h time point was selected to capture a measurable immune response to IRI while remaining within the maximum duration of anesthesia and mechanical ventilation. The 72 h time point was chosen to assess the lung at a stage when severe PGD has the greatest prognostic significance in clinical lung transplantation. Both groups underwent the same anesthesia protocols as with donor pigs. The pigs in the 4hIRI group were sacrificed via exsanguination following 4 h of reperfusion, while under anesthesia, through the ligation and transection of the inferior vena cava. The 72hIRI group pigs were weaned off anesthesia and extubated, and received immediate postoperative care, including pain management with buprenorphine (0.01–0.05 mg/kg i.v. every 6 h) and a drug regimen with immunosuppression and prophylaxis for infection and thromboembolic events, as previously described [5]. Immunosuppression treatment included methylpredinose (1 mg/kg/dose) and cyclosporin (10 mg/kg/dose). At 72 h post-transplant, the pigs were sedated and sacrificed using the same technique as described in the donor operation. Arterial blood gases were obtained from arterial line blood samples and analyzed using a RapidPoint 500 blood gas analyzer (Siemins, Munich, Germany). No pigs in this study died before reaching either sacrifice timepoints.

The control group included pigs with no lung transplant performed (Control, n = 6) (Fig 1C) which were sedated and sacrificed using the same techniques as described in the donor operation. From all groups, the left lower lobe was collected. Bronchoalveolar lavage (BAL) was collected according to standard collection protocols from a consensus document for human BAL collection [27]. In short, two instillations of 50 ml saline aliquots were performed in the right middle lobe of the right donor lung, with immediate aspiration after each instillation. Additional spleen samples, 4 cm^3^ in size, were collected from the Control group.

### Tissue processing

Porcine lung samples approximately 4 cm^3^ in size was cut and processed into single cell suspensions (detailed in the Supplemental Text). In short, tissue was minced, mixed with enzymes and dissociated with gentleMACS™ Octo Dissociator (Miltenyi Biotec, Cologne, Germany), filtered through a 70 μm cell strainer and red blood cells (RBCs) were lysed using ammonium chloride RBC lysis buffer. Cells were resuspended with phosphate-buffered saline (PBS) with 2% volume/volume fetal bovine serum and 2 mM EDTA (FACS buffer). BAL samples were centrifugated at 400 RCF for 5 mins to pellet cells and resuspended in FACS buffer. Spleen samples were minced and filtered through a 70 μm cell strainer, RBCs lysed with ammonium chloride RBC lysis buffer, and samples were resuspended in FACS buffer. Cell counts and viability were determined by hemocytometer counts with trypan blue exclusion to acquire pre-cryopreservation cell counts. All cell samples were resuspended with freezing media (heat-inactivated FBS + 10% dimethyl sulfoxide) and cryopreserved.

### Flow cytometry panel development

Novel porcine flow cytometry panels were developed to identify general lymphoid and myeloid immune cells (T cells, B cells, monocytes, macrophages, neutrophils, dendritic cells (DCs)) and their subsets. Details on how the antibodies were chosen are described in the Supplemental Text. In short, commercial antibodies made to be reactive to pig cells were first considered, followed by antibodies that were reported to be cross-reactive to porcine cells. A full list of the selected antibodies is included in Table 1. A summary of the optimization steps taken in developing the porcine flow cytometry panels is described in the Supplemental Text (Fig S1). To summarize, antibodies were first tested in a single stain using the manufacturer’s recommended concentration with either porcine spleen, peripheral blood mononuclear cells (PBMCs), or lung samples to assess functionality. Mouse spleen or PBMC samples and human PBMC samples were used as controls, depending on whether the tested antibody was cross-reactive to murine or human cells. If a positive signal was detected as expected, individual antibodies were then tested on thawed cryopreserved porcine cells and compared to fresh single cell suspensions, to assess for any artefacts that may occur from cryopreservation. If no artefacts were observed, the antibody was then titrated. Following titration of all antibodies in a panel, a multicolor stain pilot was then performed to assess issues regarding fluorescence spillover, high background spread of fluorescent signal, or high autofluorescence. If no issues were found, the panel was then determined to be optimized for use.

**Table 1:** Flow cytometry antibodies.

Antibody	Clone	Isotype	Species	Fluorochrome	Dilution	Supplier	Catalog number
Myeloid cell panel							
CD2	RPA-2.10	IgG1	Mouse	PE-Cy5	1:2	US Biological	214602
CD3	BB23-8E6-8C8	IgG2a, κ	Mouse	PerCp-Cy5.5	1:5	BD Biosciences	561478
CD16	FcG7	IgG1, κ	Mouse	Biotinylated	1:100	BD Biosciences	551395
CD45	K252.1E4	IgG1	Mouse	AF647	1:5	BioRAD	MCA1222A647
CD79a	HM47	IgG1, κ	Mouse	APC-eF750	1:100	ThermoFisher	47-0792-42
CD163	2A10/11	IgG1	Mouse	PE	1:50	BioRAD	MCA2311PE
SLA-II DR	2E9/13	IgG2b	Mouse	FITC	1:25	BioRAD	MCA2314F
6D10	6D10	IgG2a	Mouse	n/a	1:200	BioRAD	MCA2599GA
Dendritic cell panel							
CD1	76-7-4	IgG2a, κ	Mouse	Biotinylated	1:100	Novus Biologicals	NBP1–28223
CD3	BB23-8E6-8C8	IgG2a, κ	Mouse	PerCP-Cy5.5	1:5	BD Biosciences	561478
CD4	74-12-4	IgG2b, κ	Mouse	PE-Cy7	1:10	BD Biosciences	561474
CD14	433423	IgG2b	Mouse	AF700	1:5	R&D Systems	FAB4597N
CD45	K252.1E4	IgG1	Mouse	AF647	1:5	BioRAD	MCA1222A647
CD79a	HM47	IgG1, κ	Mouse	APC-eF750	1:100	ThermoFisher	47-0792-42
CD172a	BL1H7	IgG1	Mouse	Pure	1:50	BioRAD	MCA2312GA
CD204a	2F8	IgG2b	Rat	PE	1:50	ThermoFisher	MA5–16496
T cell panel							
CD3	BB23-8E6-8C8	IgG2a, κ	Mouse	PerCP-Cy5.5	1:5	BD Biosciences	561478
CD4	74-12-4	IgG2b, κ	Mouse	PE-Cy7	1:10	BD Biosciences	561473
CD8a	76-2-11	IgG2a, κ	Mouse	PE-Cy5	1:10	biorbyt	orb1251580
CD25	K231.3B2	IgG1, κ	Mouse	n/a	1:60	BioRAD	MCA1736GA
CD44	IM7	IgG2b, κ	Rat	BV605	1:30	BioLegend	103049
CD45	K252.1E4	IgG1	Mouse	AF647	1:5	BioRAD	MCA1222A647
CD45RA	MIL13	IgG1	Mouse	FITC	1:30	BioRAD	MCA1751F
CD62L	SK11	IgG2a, κ	Mouse	BV711	1:2	BD Biosciences	565040
Foxp3	FJK-16s	IgG2a, κ	Rat	AF700	neat	invitrogen	56-5773-82
B cell panel							
CD2a	RPA-2.10	IgG1	Mouse	PE	1:5	ThermoFisher	12-0029
CD21	BB6-11C9.6	IgG1, κ	Mouse	Biotinylated	1:10	Southern Biotech	4530−08
CD45	K252.1E4	IgG1	Mouse	AF647	1:5	BioRAD	MCA1222A647
CD62L	SK11	IgG2a, κ	Mouse	BV711	1:2	BD Biosciences	565040
CD79a	HM47	IgG1, κ	Mouse	APC-eF750	1:100	ThermoFisher	47-0792-42
CD80	16-10A1	IgG	Armenian Hamster	PE/Cy7	1:2	ThermoFisher	25-0801-82

BD Biosciences, San Jose, CA, United States

BioRAD, Hercules, CA, United States

biorbyt, Cambridge, United Kingdom

Invitrogen, Waltham, MS, United States

Novus Biologicals, Centennial, CO, United States

R&D Systems, Minneapolis, MN United States

Southern Biotech, Birmingham, AL, United States

ThermoFisher, Waltham, MA, United States

US Biological, Salem MA, United States

### Flow cytometry

Cryopreserved lung, BAL and spleen samples were rapidly thawed at 37 °C and stained with viability dye, then subsequently stained with antibodies directed at extracellular (membrane) proteins. Cells were fixed using either the eBioscience™ Foxp3/Transcription Factor Staining Buffer Set (Invitrogen, Waltham, MA, United States) or the BD Cytofix/Cytoperm kit (BD Biosciences, San Jose, CA, United States), depending on whether the panel contained transcription factor antibodies, and then stained with intracellular antibodies. Stained samples were run on a LSR II flow cytometer (BD Bioscience). Flow cytometry data was acquired via FACSDiva™ (BD Biosciences) and analyzed using FlowJo™ (v10.8, BD Biosciences). All samples analyzed and reported in this study represent independent biological replicates.

### Validation experiments

To validate the flow cytometry results, single cell suspensions from three Control lungs were used to create cytospin slides. Slides were stained and analyzed using cell differential counts (see Supplementary Text). mRNA extraction was performed on a cohort of the initial 72IRI group samples: lung (n = 6), BAL (n = 4) and spleen (n = 4). mRNA underwent reverse transcription-quantitative PCR (RT-qPCR) with custom pig primers targeting *PPIA*, *CD3E* and *CD79A* (see Supplementary Text).

### Statistical analysis

Data was analyzed with Prism 8 (GraphPad Software Inc, San Diego, CA, United States) and expressed as mean ± standard deviation (SD). Differences between groups were assessed by Kruskal–Wallis test when appropriate. Significance was determined as p < 0.05. No outliers were excluded.

## Results

### Immunophenotyping of major porcine immune cells and subsets

**Fig 2:**
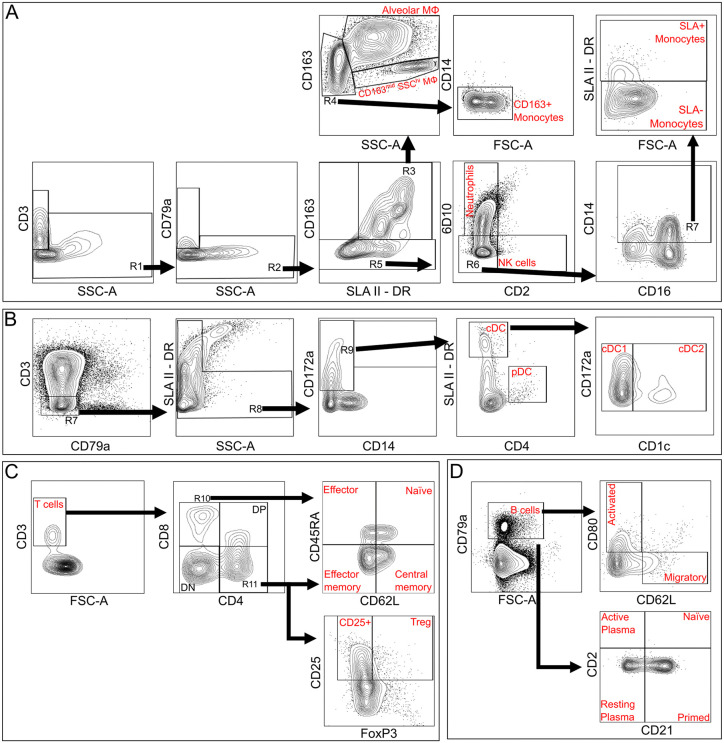
Gating strategy for flow cytometric analysis, gated on live CD45 + cells. **(A)** Myeloid panel. CD3^+^ and CD79^+^ cells were excluded (R1 and R2). CD163^+^ SLA-II-DR^+^ cells were gated (R3) and SSC^hi^ cells were considered MΦs and were subdivided using CD163 to separate into CD163^hi^SSC^hi^ Alveolar MΦ or CD163^mid^SSC^hi^ MΦ. SSClo cells were further gated based on CD14 expression (R5) and CD14^-^ cells were identified as CD163^+^ monocytes. CD163^-^ cells (R5) were subdivided using 6D10 and CD2 to identify 6D10^+^ Neutrophils, CD2^+^ NK cells and double negative cells (R6). CD14 expression was used to identify monocytes (R7) which were subdivided using SLA-II-DR to SLA-II^+^ and SLA-II^-^ monocytes. MΦ: macrophage. NK cells: natural killer cells. SLA-II-DR: Swine leukocyte antigen type II subtype DR. **(B)** DC panel. CD3^+^ and CD79^+^ cells were excluded (R7), and cells with high SLA-II-DR and SSC expression were gated (R8). CD172a was used to identify DCs (R9) which were subdivided to CD4^-^ cDC and CD4^+^ pDC. cDCs were further subdivided into CD1^-^ cDC1 and CD1^+^ cDC2. DC: dendritic cell. cDC: conventional dendritic cell. cDC1: type 1 conventional dendritic cell. cDC2: type 2 conventional dendritic cell. pDC: plasmacytoid dendritic cell. **(C)** T cell panel. CD3^+^ T cells were identified and subdivided into CD4^+^ (R10), CD8^+^ (R11), CD4^-^CD8^-^ double negative, and CD4^+^ CD8^+^ double positive populations. Both R10 and R11 were further subdivided using CD62L and CD45RA into CD62L^+^CD45RA^+^ naïve, CD62L^+^CD45RA^-^ central memory, CD62L^-^CD45RA^-^ effector memory and CD62L^-^CD45RA^+^effector T cells. R11 was also assessed for CD25 activation marker and Foxp3 transcription factor to identify activated CD4^+^CD25^+^Foxp3^-^ T cells and CD4CD25Foxp3^+^ Tregs. Treg: regulatory T cell. **(D)** B cell panel. CD79^+^ B cells were identified and subdivided using CD2 and CD21 expression to identify CD2^+^ CD21^+^ naïve and CD2^-^CD21^+^ primed B cells, or CD2^-^CD21^-^ resting and CD2CD21^-^ active plasma cells. B cells were also assessed for CD80 activation marker and CD62L migratory marker.

Our flow cytometric panels identified key immune cell populations in porcine tissues based on antigens outlined in Table 1, as well as high or low side scatter (SSC^hi^ or SSC^lo^). Pre-gating was performed for all panels to remove debris (FSC-A/SSC-A), exclude doublets (FSC-W vs. FSC-H, SSC-W vs. SSC-H), select live cells and identify CD45 + leukocytes (Fig S2A). The myeloid panel (Fig 2A) identified neutrophils (CD163^-^SLA-II-DR^-^6D10^+^), NK cells (CD3^-^CD79α^-^SLA-II-DR^-^CD2^+^), class II swine leukocyte antigen (SLA-II)^+^ and SLA-II^-^ monocytes (CD163^-^CD14^+^), CD163^+^ monocytes (CD163^+^CD14^-^SLA-II-DR^+^SSC^lo^), and two macrophage subsets: AMΦ (CD163^hi^SLA-II-DR^+^SSC^hi^) and CD163^mid^SSC^hi^ macrophages (CD163^mid^MΦ) (CD163^mid^SLA-II-DR^+^SSC^hi^). The DC panel (Fig 2B) distinguished plasmacytoid DC (pDC) (CD172^+^SLA-II-DR^+^CD4^+^) and conventional DC (cDC) (CD172^+^SLA-II-DR^+^CD4^-^) which were further subdivided to type I conventional DC (cDC1) and type II conventional DC (cDC2) based on CD1 expression. The T cell panel (Fig 2C) identified CD4^+^, CD8^+^, CD4^+^CD8^+^ double positive and CD4^-^CD8^-^ double negative T cells. Within the CD4^+^ and CD8^+^ T cell populations, naïve (CD45RA^+^CD62L^+^), effector (CD45RA^+^CD62L^-^), effector memory (CD45RA^-^CD62L^-^) and central memory (CD45RA^-^CD62L^+^) subsets were identifiable. Regulatory T cells (Treg) (CD25^+^Foxp3^+^) were identified within CD4^+^ T cells. The B cell panel (Fig 2D) identified B cells (CD79α^+^) and their subsets: activated (CD80^+^), migratory (CD62L^+^), naïve (CD2^+^CD21^+^), primed (CD2^-^CD21^+^), resting plasma (CD2^-^CD21^-^) and active plasma cells (CD2^+^CD21^-^). Gates were determined using fluorescence-minus-one (FMO) controls (S2B-E Fig).

### Validation of porcine-specific flow cytometry panels

**Fig 3:**
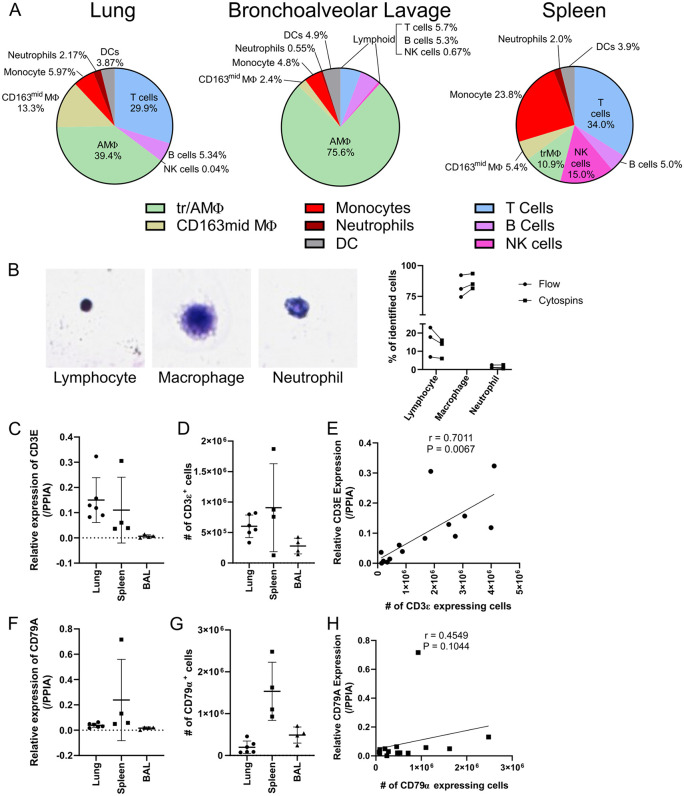
Validation of porcine flow cytometry. **(A)** Summary of major immune cells identified by flow cytometry in lung (n = 6), bronchoalveolar lavage (BAL) (n = 4) and spleen samples (n = 6). DC: dendritic cells. NK: natural killer. MΦ: macrophage. tr/AMΦ: tissue resident/alveolar macrophage. **(B)** (Left) Representative photomicrographs of Kwik-Diff™ stained lymphocyte, macrophage and neutrophil. (Right) Comparison of the proportion of lymphocytes, macrophages and neutrophils in BAL (n = 3), identified by flow cytometry (circle) and cytospin counts (square). **(C)** Relative expression of porcine CD3E transcripts from lung (n = 6), spleen (n = 4) and BAL (n = 4) samples. Data was presented as mean ± SD. **(D)** Number of CD3ε+ cells from matched lung, spleen and BAL samples by flow cytometry. Data was presented as mean ± SD. **(E)** Correlation of relative transcript expression and number of cells expressing CD3ε for matched samples. Spearman’s rank correlation test performed and reported as “r =”, with p-value reported as “P =”. **(F)** Relative expression of porcine CD79A transcripts from lung (n = 6), spleen (n = 4) and BAL (n = 4) samples. Data was presented as mean ± SD. **(G)** Number of CD79α+ cells from matched lung, spleen and BAL samples by flow cytometry. Data was presented as mean ± SD. **(H)** Correlation of relative transcript expression and number of cells expressing CD79α for matched samples. Spearman’s rank correlation test performed and reported as “r =”, with p-value reported as “P =”.

Flow cytometric analysis of porcine lung, BAL and spleen tissues revealed distinct cell compositions (Fig 3A). Lungs were dominated by myeloid cells, specifically a mixture of AMΦ and CD163^mid^MΦ, whereas BAL primarily contained AMΦ. The lack of CD163^mid^MΦ in BAL indicates this population resides outside the alveolar space, suggesting they are pulmonary intravascular macrophages, a population reported to be in pig lungs [28]. Spleen tissue contained abundant lymphoid cells and monocytes, consistent with the tissue’s role as a secondary lymphoid organ.

To validate our flow-based cell identification, single cell suspensions from 3 BAL samples underwent both flow cytometry staining and differential cell counting following cytospin protocols. Proportions of lymphocytes, macrophages and neutrophils were similar between both flow and cytospin data (Fig 3B). To further assess the reliability of flow-based lymphocyte phenotyping, RT-qPCR was performed assessing *CD3E* and *CD79A* transcripts in single cell suspensions from lung, BAL and spleen (Figs 2C and F). The same suspensions underwent flow cytometry to measure CD3ε and CD79α protein expression (Figs 2D and G). Significant correlation was observed between CD3E transcript and CD3ε protein expression, with CD79A and CD79α comparisons trending to significance (Figs 2E and H). Notably, the variability of T and B cell proportions were higher in the porcine spleen than in lung or BAL, which may be due to sampling variation, as a limited section of the spleen was processed and variability in the relative representation of red and white pulp may change across small samples and may have affected the number and composition of immune cells in the resulting single cell suspension.

### Peripheral myeloid cells dominate the immune response to IRI

**Fig 4:**
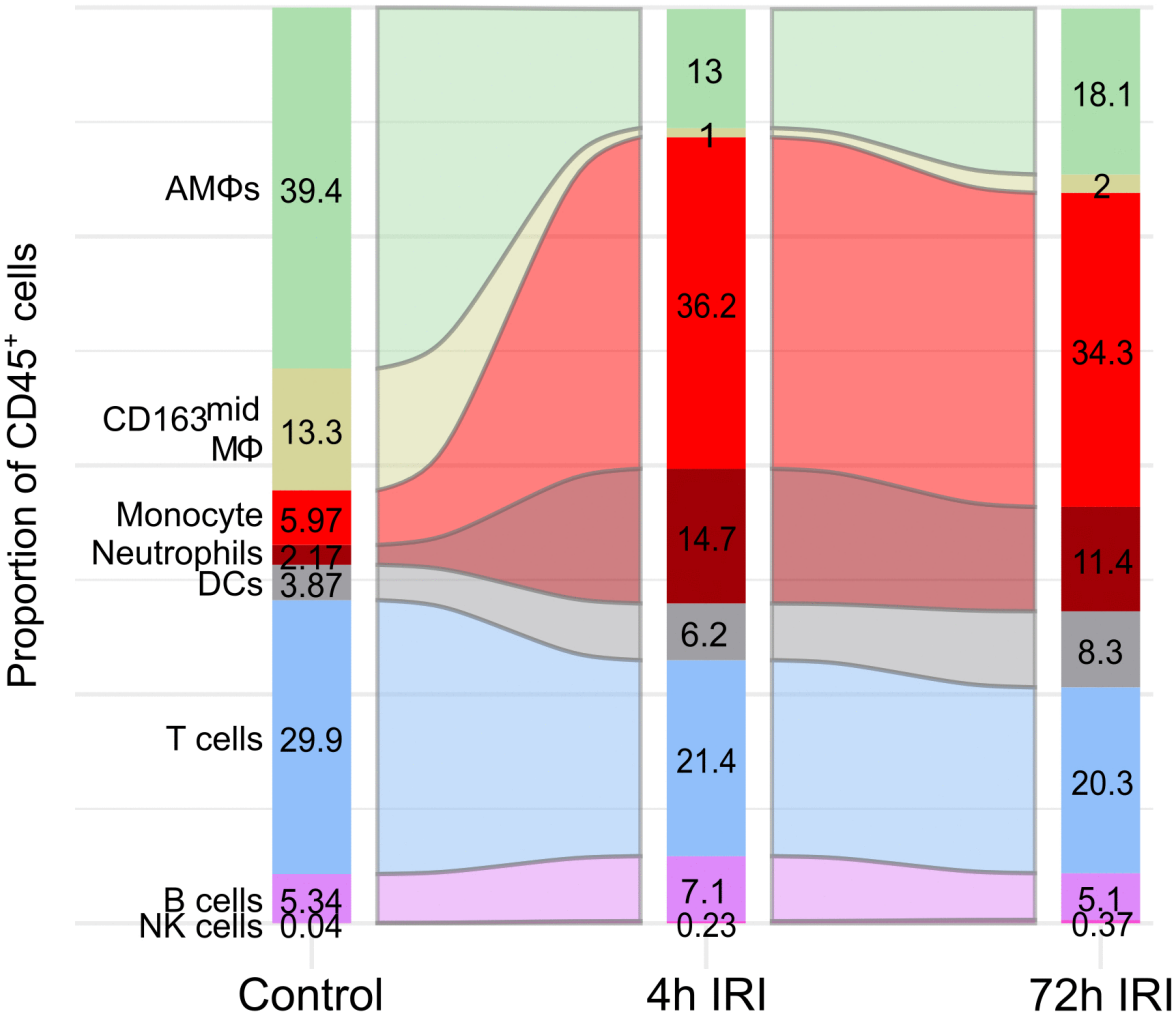
Changes in leukocytes between Control, 4hIRI and 72hIRI lung samples. Percentages reported as a proportion of CD45 + cells. AMΦ: alveolar macrophage. DC: dendritic cell. MΦ: macrophage. NK cells: natural killer cells.

Using our optimized porcine flow cytometry panels, pulmonary immune cell composition was characterized at baseline (Control, n = 6), immediately following reperfusion (4hIRI, n = 7) and three days after reperfusion (72hIRI, n = 6). The majority of leukocytes at baseline were of myeloid origin, specifically resident macrophages. AMΦ and CD163^mid^MΦ formed 52.7% of all immune cells in Control lungs (Fig 4). Following IRI, the proportion of the lung resident macrophages were displaced by neutrophils and monocytes at 4hIRI lungs and 72hIRI lungs (Fig 4). Lymphoid cells made up a minority of cells in all groups and were mostly composed of T cells (Fig 4).

**Fig 5:**
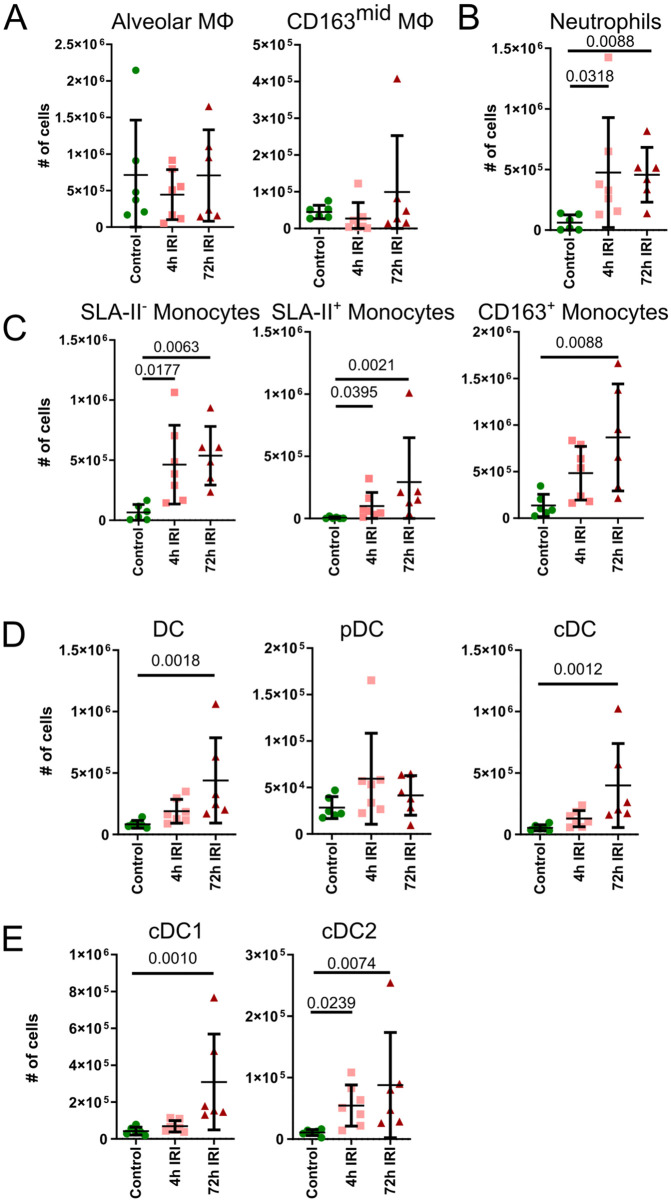
Changes in myeloid cells between Control, 4hIRI and 72hIRI lung samples, reported as absolute counts. **(A)** Resident macrophages (MΦ): (Left) Alveolar MΦ and (Right) CD163^mid^MΦ. **(B)** Neutrophils. **(C)** Monocytes: (Left) SLA-II^-^ monocytes, (Center) SLA-II^+^ monocytes, (Right) CD163^+^ monocytes. **(D)** Dendritic cells (DC): (Left) All DCs, (Center) plasmacytoid DCs (pDCs), (Right) conventional DCs (cDCs). (E) cDC subsets: (Left) cDC1, (Right) cDC2. For all plots, n = 6 for Control and 72hIRI, n = 7 for 4hIRI. Data is presented as mean ± SD and compared using Kruskal-Wallis test followed by Dunn’s multiple comparisons with Bonferroni correction. Adjusted P values reported if < 0.05.

To determine whether changes in proportions reflect true expansions or contractions in cell numbers, the absolute number of immune cells were calculated by extrapolating manual hemocytometer counts using percentages of live cells determined by flow cytometry. While the mean lung cell numbers increased gradually from Control to 72hIRI group, this increase was not statistically significant (Fig S3A). AMΦ and CD163^mid^MΦ had little change between Control, 4hIRI and 72hIRI lungs (Fig 5A), indicating that their numbers were not significantly impacted by IRI. Next, the immune cells from the periphery were assessed. The number of neutrophils significantly increased from Control to 4hIRI lungs but changed little between 4hIRI and 72hIRI lungs (Fig 5B). Among the monocyte subsets, SLA-II^-^ and SLA-II^+^ monocytes, followed a similar pattern with neutrophils, significantly increased from Control to 4hIRI lung and had no further changes between 4hIRI and 72hIRI lungs (Fig 5C, left and middle panel). This contrasts with the CD163^+^ monocytes, which had a modest but non-significant increase from Control to 4hIRI lungs, but then significantly increased in the 72hIRI group (Fig 5B right panel). Consistent with the flow cytometric analyses, a quantitative increase in neutrophils and monocytes was observed in representative hematoxylin-eosin-stained formalin-fixed paraffin embedded lung tissue sections (S3B Fig). DCs were also augmented at 72 h (Fig 5D left panel). Among the DC subsets, cDCs primarily accounted for the observed increase, whereas pDCs remained relatively unchanged across the three groups (Fig 5D, middle and right panel). cDCs were next classified into cDC1 and cDC2 subsets. Both subsets were significantly higher in 72hIRI lungs (Fig 5E). Notably, cDC2s increased in 4hIRI lungs, whereas cDC1s were higher in the 72hIRI lungs (Fig 5E). When the absolute counts of myeloid cells were compared to the P/F ratio of all 72h samples, neutrophils and the CD163^+^ monocyte subset had a significant inverse correlation (Fig S3C-D). Altogether the myeloid populations exhibited the largest changes within pulmonary immune cells, with peripheral myeloid populations significantly increased in both 4 h and 72 h post-reperfusion, and little change in the absolute numbers of resident macrophages and pDCs.

### A transient CD4 + T cell response is involved in early IRI

**Fig 6:**
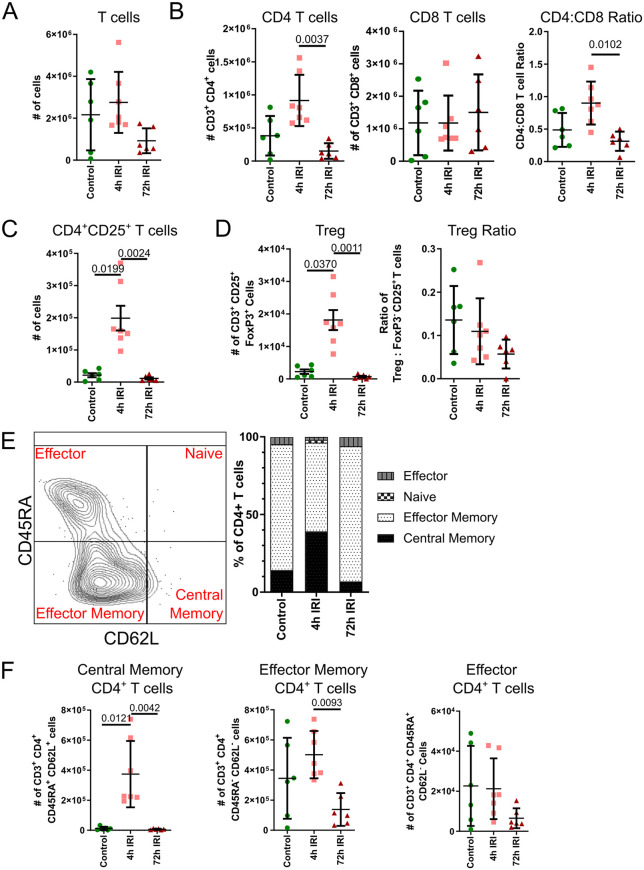
Changes in T cells between Control, 4hIRI and 72hIRI lung samples, reported as absolute counts. **(A)** T cells. **(B)** (Left) CD4^+^ T cells. (Center) CD8^+^ T cells. (Right) CD4 to CD8 T cell ratio. **(C)**. CD25CD4^+^ T cells. **(D)** (Left) T regulatory (Treg) cells. (Right) Treg to CD4CD25Foxp3^-^ T cells ratio. **(E)** (Left) Representative flow cytometry plot of CD62L x CD45RA, gated on CD4^+^ T cells. (Right) Stacked bar plot of effector naïve, effector memory and central memory subsets as a proportion of CD4^+^ T cells, in Control, 4hIRI and 72hIRI lung samples. **(F)** (Left) Central memory CD4^+^ T cells. (Center) Effector memory CD4^+^ T cells. (Right) Effector CD4^+^ T cells. For all plots, n = 6 for Control and 72hIRI, n = 7 for 4hIRI. Data is presented as mean ± SD and compared using Kruskal-Wallis test followed by Dunn’s multiple comparisons with Bonferroni correction. Adjusted P values reported if < 0.05.

T cells made up a sizable proportion of pulmonary immune cells in all sample groups and therefore were investigated in detail. T cells were not significantly different between the groups, exhibiting only a non-significant decrease in numbers by 72 h (Fig 6A). Dividing T cells to CD4^+^ and CD8^+^ subsets, we observed an increase in CD4^+^ T cells at 4 h, which fell significantly at 72 h (Fig 6B, left panel), while CD8^+^ T cells numbers remained stable across the three groups (Fig 6B, middle panel). The ratio of CD4^+^ to CD8^+^ T cells were assessed, and Control lungs favored CD8^+^ T cells, albeit with wide variance, which may reflect the pig’s prior exposure to viral pathogens encountered in farm environments (Fig 6B right panel). In 4hIRI lungs, the ratio increased to 0.9 CD4^+^:CD8^+^ T cells, reflecting the infiltration of CD4^+^ T cells into the lung. By 72 h, the ratio returned to favor CD8^+^ T cells (Fig 6B right panel).

Activated CD25^+^CD4^+^ T cells were also specifically increased in 4hIRI lungs, with approximately 10 times more cells compared to Control or 72hIRI lungs (Fig 6C). Tregs – defined here as CD4^+^CD25^+^Foxp3^+^ T cells – were also significantly increased in 4hIRI lungs compared to other groups (Fig 6D, left panel), however the ratio of Tregs to activated CD4^+^CD25^+^Foxp3^-^ T cells was not significantly different between the groups (Fig 6D, right panel). CD4^+^ T cell subsets – Naïve, central memory, effector memory and effector CD4^+^ T cells were identified using CD62L and CD45RA. Naïve CD4^+^ T cells were not present in appreciable amounts, constituting 0.22%, 1.73 and 0.12% of all CD4^+^ T cells in Control, 4hIRI and 72hIRI lungs respectively (Fig 6D). Both memory subsets of CD4^+^ T cells were numerically higher in 4hIRI lungs, with central memory CD4^+^ T cells significantly higher in 4hIRI lungs than in Control or 72hIRI lungs (Fig 6F, left panel) and effector memory CD4^+^ T cells higher in 4hIRI lungs than in 72hIRI lungs (Fig 6F, center panel). Effector CD4^+^ T cells were not statistically different between the three groups, though there appeared to be a reduction in these cells in 72hIRI lungs (Fig 6F, right panel). B cells had no significant changes in proportions or numbers at the timepoints assessed (S4 Fig), suggesting that any deviations in these populations resulting from IRI would have occurred at timepoints other than 4 h and 72 h. Altogether, little infiltration of lymphoid cells occurred in our models but specific subpopulations of CD4^+^ T cells were increased in numbers at the 4 h timepoint in a transient manner.

## Discussion

In this study, we characterized the immune cell dynamics following IRI in two porcine orthotopic lung transplant models, using novel flow cytometry panels of pig-specific antibodies. Within the first 4 h after porcine lung reperfusion, a significant increase neutrophils and monocytes was observed, which is presumed to be an influx from the peripheral immune system based on the rapidity of the increase. A transient CD4^+^ T cell response was also observed within 4 h post-reperfusion. Following 72 hours, there was a significant increase in CD163^+^ monocytes and DCs, indicating a maturing inflammatory response. To our knowledge, this is the first report of immune cells infiltration and activation in a porcine orthotopic lung transplant model. It provides an opportunity to compare the IRI immune response at 4 h versus 72 h post-reperfusion, and against other animal models and human lung grafts.

Most of our understanding of lung IRI mechanisms comes from rodent studies. Donor non-classical monocytes (F4/80^+^Ly6C^lo^MHCII^-^ intravascular monocytes) play a significant role in recruiting recipient monocytes [16,29] and activating AMΦ [17] both of which mediate IRI through neutrophil extravasation into the lung [30]. However, mouse monocytes differ more from human monocytes than pig monocytes [31], necessitating validation of these findings within a porcine model. Despite their similarities, porcine monocytes do have notable differences from humans, such as a lacking a distinct CD14^-^CD16^+^ nonclassical monocyte population [31]. A comparative analysis of porcine monocytes reported four subsets which follow a maturation pathway: (I) CD14^+^CD163^-^SLA-DR^-^, (II) CD14^+^CD163^-^SLA-DR^+^, (III) CD14^+^CD163^+^SLA-DR^+^, and (IV) CD14^-^CD163^+^SLA-DR^+^ subset [31,32]. In our study, we identified three of these subsets: CD14^+^CD163^-^SLA-DR^-^ monocytes (SLA-II^-^ monocytes), CD14^+^CD163^-^SLA-DR^+^ monocytes (SLA-II^+^ monocytes), and CD14^-^CD163^+^SLA-DR^+^ monocytes (CD163^+^ monocytes). We observed that SLA-II^-^ monocytes are present in the lung at steady state, whereas SLA-II^+^ monocytes appear only during IRI, This suggests a homeostatic role for SLA-II^-^ monocytes similar to donor non-classical monocytes in rodent models [29], though further phenotyping is required to confirm donor origin. Both SLA-II^-^ and SLA-II^+^ monocytes increased from baseline to 4 h post-reperfusion, with no significant change between 4 h and 72 h post-reperfusion. In contrast, CD163^+^ monocytes significantly increased only at 72 h, indicating a maturing inflammatory response. Notably, porcine CD163^+^ monocytes resemble human intermediate CD14^+^CD16^+^ monocytes [31]. Intermediate monocytes have not been reported as a significant population in rodent IRI due to difficulty distinguishing them from non-classical monocytes [33], highlighting the strength of using a porcine model. Altogether, our porcine model confirms key findings from rodent models of IRI and identifies CD163^+^ monocytes as a monocyte subset involved in the later timepoints, that may be a potential therapeutic target.

Activated AMΦ play a critical role in IRI through pro-inflammatory cytokines and chemokines such as IL-8 and chemokine C-C motif ligand 2 (CCL2) [14,17]. While we observed no differences in AMΦ cell numbers, it is possible that AMΦ undergo functional changes during IRI not captured by our flow cytometry panels. Further work is needed to characterize the functional properties of these cells in a porcine model.

Few studies have investigated the role of DCs in lung IRI. In other organs, DCs appear to be either protective or harmful depending on the subset [34]. In the liver, cDCs secrete anti-inflammatory IL-10 which reduces IRI [35], whereas pDCs mediate IRI through type I interferons in a cardiac transplant study [36]. pDCs can rapidly produce type I interferons. In contrast, cDCs, commonly differentiated from monocytes, act as potent antigen-presenting cells and can be further divided into two subsets, based on transcription factor expression: cDC1 and cDC2 [37]. In our porcine model, DCs were identified by CD172a-positivity and absence of CD14, with CD4 expression used to distinguish pDCs from cDCs [38]. Porcine cDCs were further subdivided based on CD1 expression to identify cDC1 and cDC2 [39]. cDC1 can perform pro-inflammatory functions, such as antigen presentation and priming of T helper 2 and T helper 17 differentiation [37], as well as homeostatic functions, including IL-10 production and Tregs induction [40]. In a mouse orthotopic lung transplant model, donor and recipient-derived cDC1s play contrasting roles, with recipient-derived cDC1s promoting acute and chronic lung rejection [41]. cDC1 is also implicated in allograft rejection in other organs; for example, clinical samples from upper extremity skin transplant recipients showed enrichment and activation of cDC1s in allograft skin compared to native skin, while pDCs and cDC2s showed no differences [42]. In our model, cDC1 numbers increased at 72 h post-reperfusion, likely originating from recipient monocytes given the concurrent rise in peripheral monocytes. This increase highlights the maturation of the immune response between 4 h and 72 h post-reperfusion. cDC2 was a minimal population in controls, but increased at both 4 h and 72 h post-reperfusion. cDC2 specializes in producing pro-inflammatory cytokines and chemokines [43], consistent with its expansion during IRI. These findings suggest that both cDC1 and cDC2 initially respond in IRI, potentially originating from recipient monocytes. While cDC2 numbers may decline after IRI resolution, cDC1s may persist and contribute to allograft rejection. Altogether, this is the first analysis of porcine DCs in pulmonary IRI and demonstrates similarities to IRI studies in other organs using rodent models.

Lymphoid cells contributions to IRI have received less attention than myeloid cells, yet evidence suggests the adaptive immune system plays a role in IRI and PGD development. In a mouse hilar clamp model, CD4^+^ T cells accumulate in the lung after reperfusion and their depletion attenuates IRI [10]. Similarly, in a rat orthotopic lung transplant model, CD4^+^ T cell infiltrate and become activated in the graft at 12 h post-transplant, as reflected by increased CD25 expression [44], highlighting their short-term involvement in IRI. Our study expands on these observations by assessing T cell population at 72 h post-transplant. Interestingly, our results show no significant change in T cells numbers within the lung allograft at either 4 h and 72 h. In the aforementioned rat study, infiltrating recipient T cells peaked at 1 h and returned to baseline within 3 h [44]. This study also reported that the proportion of CD4^+^CD25^+^ T cells remains unchanged at 2 h post-reperfusion but increased significantly by 12 h. In our study, CD4^+^CD25^+^ T cells were higher at 4 h but returned to control levels by 72 h, suggesting that the activated CD4^+^ T cells represents a transient response to IRI. This phenomena was also reported in a mouse model of ischemic liver [45]. While few studies have examined activated CD4^+^ T cells in lung IRI, research in other solid organ transplant models indicate that, through antigen-independent mechanisms, these cells promote a pro-inflammatory environment by secreting IL-17 and interferon-γ [46–48]. CD4^+^ effector and memory T cells are subsets most associated with IRI in other organs [49–51]. Similarly, we observed a transient increase in both central and effector memory CD4^+^ T cells in 4hIRI lungs. These T cells likely represent a population of tissue-resident memory T cells that rapidly respond to IRI; however, confirmation would require the inclusion of residence markers in the flow panel, such as CD49a, CD69 or CD103. Notably the transient increase was also observed for Tregs, suggesting a pan-CD4 + reduction that is associated with the 72hIRI group. This reduction may reflect the immunosuppression administered only to the 72hIRI group to closely mimic clinical post-operative regimens. Cyclosporine is known to inhibit CD4^+^ T cell activation and proliferation by blocking TCR signaling [52], and Tregs are particularly susceptible to cyclosporin [53], which may explain the reduced Treg:T cell ratio we observed. Because this transient response may be influenced by immunosuppression, it remains unclear whether activated CD4^+^ T cells play a specific role in pulmonary IRI pathogenesis. Further studies are needed to characterize this response.

While our study has provided valuable insights into immune cell changes in pulmonary IRI using a porcine model, several limitations must be acknowledged. First, there is phenotypic overlap in several myeloid populations, particularly monocytes and DCs. Although we used CD14 expression to exclude monocytes from our DC analysis, this approach limited our ability to identify other DC subsets, such as monocyte-derived DCs which share markers from both populations. The lack of established protein markers to distinguish these populations in pigs remain a major challenge. Second, by relying solely on surface protein characterization, we were unable to fully characterize the functional properties of several immune cell populations. The function of AMΦ and T cells could be further elucidated through cytokine analysis and *in vitro* functional assays. Third, the absence of a sham surgery control introduces ambiguity as to whether the observed results stem from IRI or surgical stress. Surgical stress induces an acute systemic inflammatory response characterized by increased blood monocytes, neutrophils, and inflammatory cytokines [54]. However, in sham surgeries, systemic monocytes and neutrophils returned to baseline levels by 72 h post-surgery [54], and our analysis focused on lung leukocytes, which reflects local IRI responses than systemic effects. Despite these limitations, our findings contribute to deeper understanding of lung IRI in a model that closely reflects the clinical conditions and provides a foundation for future functional studies.

To conclude, using novel porcine-specific flow cytometry panels we observed infiltrating myeloid cells and changes in lymphoid cell activation shortly after reperfusion and three days post-transplant in a porcine orthotopic lung transplant IRI model. Our findings align closely with reports from rodent models, reinforcing the validity of the porcine IRI model and offering a comprehensive overview of the cellular changes in lung IRI. One such change is the infiltration of CD163^+^ monocytes and cDC1s at late IRI, which may be a potential therapeutic target in future studies. These flow cytometry panels, sourced from commercially available antibodies for ease of use, add value to porcine research models by enabling more detailed immunological assessments in studies evaluating preclinical therapies for lung transplantation, or other immunological responses using a porcine model.

## Supporting information

S1 Fig Flowchart outlining the steps for flow cytometry panel development and optimization. (TIF)

S2 Fig Supplemental flow cytometry plots. (A) Pre-gating strategy used to gate on CD45^+^ live cells. (B) Fluorescence minus one (FMO) controls used for the myeloid panel. FMO: fluorescence minus one. (C) FMO controls used for the dendritic cell panel. (D) FMO controls used for the T cell panel. (E) FMO controls used for the B cell panel.
(TIF)

S3 Fig Supplemental myeloid figures. (A) Absolute counts between Control, 4hIRI and 72hIRI lung samples. (B) Qualitative histological assessment of representative hematoxylin-eosin-stained formalin-fixed paraffin embedded lung tissue sections. Red arrowheads denote neutrophils. Black arrowheads denote monocytes. (C-D) Correlation of PaO_2_/FiO_2_ ratio (P/F ratio) and number of neutrophils (C) or monocytes (D). Spearman’s rank correlation test performed and reported as “r =”, with p-value reported as “P =”. Linear regression goodness-of-fit reported as R^2^.
(TIF)

S4 Fig Changes in B cells between Control, 4hIRI and 72hIRI lung samples, reported as proportion of CD45^+^ cells and absolute counts. (A) B cells. (B) CD80^+^ activated B cells. (C) CD62L^+^ migratory B cells. For all plots, data is presented as mean ± SD.
(TIF)

S1 File Supplemental Text. (DOCX)

S1 Table Sequences of the primers used in RT-qPCR analysis. (DOCX)

## Abbreviations

AMΦ: alveolar macrophage
BAL: bronchoalveolar lavage
CCL2: chemokine C-C motif ligand 2
CD163^mid^MΦ: CD163^mid^SSC^hi^ macrophage
cDC: conventional dendritic cell
cDC1: type I conventional dendritic cell
cDC2: type II conventional dendritic cell
DC: dendritic cell
EDTA: ethylenediaminetetraacetic acid
FACS: fluorescence-activated cell sorting
HEPES: 4-(2-hydroxyethyl)-1-piperazineethanesulfonic acid
HI-FBS: heat inactivated-fetal bovine serum
IL: interleukin
IRI: Ischemia-reperfusion injury
PBMC: peripheral blood mononuclear cell
pDC: plasmacytoid dendritic cell
PGD: primary graft dysfunction
RBC: red blood cell
RT-qPCR: reverse transcription-quantitative polymerase chain reaction
SLA-II: swine leukocyte antigen, class II
Treg: regulatory T cell.
