# Supplementary material for: Characterizing the myeloid and lymphoid immune response in a porcine model of pulmonary ischemia-reperfusion injury through flow cytometry
Source: PLoS One. 2026 May 21;21(5):e0344691. doi: 10.1371/journal.pone.0344691 (PMC13193541; doi:10.1371/journal.pone.0344691)
Supplement: S1 File — (DOCX) [file pone.0344691.s005.docx]

**Supplemental Text.**

**MATERIALS AND METHODS**

*Tissue processing*

Lung tissue was minced and submerged into digestion solution containing 1.1 mg/ml of Collagenase A (Roche, Basel, Switzerland) and 0.2 mg/ml DNase I (Roche) in Hanks’ buffered salt solution plus 5% volume/volume heat-inactivated fetal bovine serum (HI-FBS) and 10 mM 2-[4-(2-hydroxyethyl)piperazin-1-yl]ethanesulfonic acid (HEPES) and dissociated with gentleMACS™ Octo Dissociator (Miltenyi Biotec, Cologne, Germany) per manufacturer’s instructions. Dissociate was strained with 70 μm cell strainer and red blood cells (RBC) lysed with RBC lysis buffer (155 mM NH_4_Cl, 10 mM KHCO_3_, 68.5 µM ethylenediaminetetraacetic acid (EDTA)). Lung single cell suspensions were resuspended with flow cytometry staining buffer (FACS buffer, phosphate-buffered saline with 2% volume/volume heat-inactivated fetal bovine serum and 2 mM EDTA) and cell counts measured using hemocytometer with trypan blue exclusion of cell viability to acquire a pre-cryopreservation cell counts

*Flow cytometry panel development*

To enhance the utility of these panels, only commercially available antibodies were employed, and were selected for their ability to identify key populations and their subsets. Antibodies made specifically to be reactive to pig cells were taken into consideration first. If no anti-pig antibody for a desired marker was identified, alternate antibodies that are reported to be cross-reactive to pigs were assessed and included if found to adequately stain porcine tissue.

Markers targeted by the flow cytometric panels were selected based on their capability to identify major myeloid and lymphoid cells, and their subsets. Initially, key markers that are present in major lymphoid or myeloid populations, such as CD3 for T cells, were selected first. Next markers to identify subsets of major lymphoid and myeloid populations were included, such as CD4, CD25, and Foxp3. A full table of the chosen antibodies is included in Table 1.

Each individual antibody was tested in a single stain with either porcine spleen, peripheral blood mononuclear cells (PBMC), or lung to assess functionality. Mouse spleen or PBMC controls were included in the stain to act as a negative or positive control dependent on whether the tested antibody was cross-reactive to murine cells. Similarly, human PBMC were used as a control. Functionality was tested by running the stained cells through a three laser LSR II flow cytometer (BD Bioscience, San Jose, CA, United States) and observing for a positive signal. If a positive signal was detected as expected, the antibody is then tested on thawed cryopreserved tissue to assess for any artifacts that may occur from cryopreservation and compared with fresh tissue. If no artefacts were observed, the antibody was then titrated. Following the titration of all antibodies in a single panel, a multicolor stain pilot was then performed to assess issues regarding fluorescence compensation, high autofluorescence, or high background spread of fluorescent signal. If no issues were found, the panel is then optimized for use. FACS buffer was used throughout the staining protocol.

*Cytospins preparation and cell differential staining*

SuperFrost™ Plus (Fisher Scientific, Waltham, MA, United States) glass slides were inserted into a Cytospin slide clip, and a cytofunnel with filter card was placed atop. Single cell suspensions were agitated and 2x10^4^ total cells were aliquoted into cytofunnel. 200 μl of 1% paraformaldehyde in FACS buffer was added into the cytofunnel, and left to incubate for 5 min. Following incubation, the slide holder was loaded into the Cytospin™ centrifuge (Thermo Fisher, Waltham MA, United States) and centrifuged at 800 rpm for 3 min. Following centrifugation, slides were released from the holder and left to air dry overnight.

Cytospin slides were stained with Erpedia™ Shandon™ Kwik-Diff stain (Fisher Scientific) according to manufacturer’s protocols. Images were scanned using an Aperio CS2 slide scanner (Leica Microsystems, Wetzlar, Germany) and viewed with Aperio ImageScope (v12.Leica)

*RNA extraction, cDNA synthesis and reverse-transcription quantitative PCR*

RNA extraction was performed on thawed cryopreserved porcine lung, spleen and BAL samples using the RNeasy Micro Kit (Qiagen, Hilden, Germany), and cDNA synthesized using the iScript Advanced cDNA synthesis kit (BioRAD, Hercules, CA, United States) according to the manufacturer’s protocols. Reverse-transcription quantitative PCR (RT-qPCR) was performed using the Sso Advanced Universal SYBR Green Supermix (BioRAD). Pig primers targeting *PPIA*, *CD3E* and *CD79A* were developed, with specific sequences reported in Table S1. Data was analyzed using CFX Maestro Software for Real-time PCR (BioRAD) and normalized to *PPIA* expression.

*Tissue Processing and Hematoxylin-Eosin (H&E) Staining*

Collected porcine lung tissue were fixed in 10% neutral buffered formalin for 24h and processed using an Excelsior AS automated tissue processor (Thermo Fisher Scientific). Tissues were embedded in paraffin to generate formalin-fixed paraffin embedded blocks.

FFPE tissue blocks were sectioned at 4 µm thickness and mounted onto glass slides. For histological analysis, tissue sections were deparaffinized in xylene and rehydrated. Slides were stained with hemotaxylin, differentiated and blued, and counterstained with eoisin. Sections were dehydrated, cleared and coverslipped using mounting medium. H&E stained slides were scanned using an Aperio CS2 slide scanner (Leica Microsystems) and viewed with Aperio ImageScope (v12, Leica).

*Statistical Analysis*

Data was analyzed with Prism 8 (GraphPad Software Inc, San Diego, CA, United States) and expressed as means ± SD. Differences between groups were assessed by Kruskal**–**Wallis test when appropriate. Significance was determined as p < 0.05.

**S1Table.** Sequences of the primers used in RT-qPCR analysis.

| **Target mRNA** | **Forward primer** | **Reverse primer** |
| --- | --- | --- |
| ***PPIA*** | CTGCTGTCTTTGGAACTTTGTC | CCACCGTCTTCTTCGACATC |
| ***CD3E*** | TATACCTGCACAGTCGGAGAG | GATTGTGACCACTGCCATCA |
| ***CD79A*** | ATCATAATCATCCTGGACATCCG | ATCATCCTGCTGATCTGTGC |
