# Supplementary material for: Characterizing the myeloid and lymphoid immune response in a porcine model of pulmonary ischemia-reperfusion injury through flow cytometry
Source: PLoS One. 2026 May 21;21(5):e0344691. doi: 10.1371/journal.pone.0344691 (PMC13193541; doi:10.1371/journal.pone.0344691)
Supplement: S1 Table — (DOCX) [file pone.0344691.s006.docx]

**S1Table.** Sequences of the primers used in RT-qPCR analysis.

| **Target mRNA** | **Forward primer** | **Reverse primer** |
| --- | --- | --- |
| ***PPIA*** | CTGCTGTCTTTGGAACTTTGTC | CCACCGTCTTCTTCGACATC |
| ***CD3E*** | TATACCTGCACAGTCGGAGAG | GATTGTGACCACTGCCATCA |
| ***CD79A*** | ATCATAATCATCCTGGACATCCG | ATCATCCTGCTGATCTGTGC |
